# FOXD3 regulates anaplastic thyroid cancer progression

**DOI:** 10.18632/oncotarget.16853

**Published:** 2017-04-05

**Authors:** Huabin Yin, Tong Meng, Lei Zhou, Feixing Zhao, Xiufang Li, Yundong Li, Mengjun Hu, Haiyan Chen, Dianwen Song

**Affiliations:** ^1^ Department of Orthopedics, Shanghai Bone Tumor Institute, Shanghai General Hospital, School of Medicine, Shanghai Jiaotong University, Shanghai, PR China; ^2^ Department of Orthopedic Oncology, Changzheng Hospital, The Second Military Medical University, Shanghai, PR China; ^3^ Zhuji People's Hospital of Zhejiang Province, Zhuji, Zhejiang, PR China; ^4^ Department of General Surgery, Haiyang People's Hospital, Haiyang, Shangdong, PR China; ^5^ Department of Rheumatology, Shanghai Guanghua Hospital of Integrated Traditional and Western Medicine, Shanghai, PR China

**Keywords:** FOXD3, human anaplastic thyroid cancer, metastasis, MAPK/ERK signal pathway

## Abstract

Anaplastic thyroid cancer (ATC) is an aggressive malignancy with poor prognosis. It was reported that Forkhead box D3 (FOXD3) transcription factor is associated with several cancers. We investigated its antitumorigenic role of ATC in this study. The ATC cell lines SW1736 and K18 exhibited lower FOXD3 expression than the Nthy-ori-3-1 normal thyroid cell line. FOXD3 downregulation in ATC cell lines promoted invasiveness and epithelial-to-mesenchymal transition (EMT) and decreased cellular apoptosis. *FOXD3* silencing also enhanced p-ERK levels in the ATC cell lines, suggesting it negatively regulated MAPK/ERK signaling. Silencing FOXD3 in SW1736 cells also led to generation of larger xenograft tumors with high p-ERK and low E-cadherin levels. Moreover, human ATC samples showed lower FOXD3 and higher p-ERK levels than samples of normal thyroid tissue. These findings demonstrate that FOXD3 acts as a tumor suppressor during anaplastic thyroid carcinogenesis and highlight its potential for clinical application.

## INTRODUCTION

Thyroid cancer is an aggressive malignancy with extremely poor survival rates. Although conventional therapeutic modalities are effective in early-stage thyroid cancer patients, they are largely ineffective in advanced stage patients with differentiated, undifferentiated/anaplastic or medullary thyroid cancers (DTC, ATC or MTC) [1–4]. Hence, there is an urgent need to discover, develop and critically evaluate novel therapeutic drugs to clinically treat thyroid cancer patients [4]. Thyroid cancers are associated with mutations in many critical genes like *BRAF, RAS*, catenin (cadherin-associated protein), beta 1, *PIK3CA, TP53, AXIN1, PTEN*, and *APC* [5]. Also, recent studies have shown that follicular, papillary and anaplastic types of thyroid cancers have tumorigenic stem cell origin [6].

Forkhead box D3 (FOXD3) is an important member of the FOX transcription factor family and is located on human chromosome 1p31 [7]. FOXD3 is critical for embryonic development, especially in maintenance of pluripotency in ESCs and regulation of neural crest formation, migration and differentiation. In addition, FOXD3 plays a critical role in tumor initiation and growth by interacting with other transcription factors like TWIST1 [8, 9]. Many studies have shown the relevance of FOXD3 in tumorigenesis. FOXD3 inhibits non-small cell lung cancer progression [7]. Its downregulation is associated with distal metastases in invasive ductal carcinomas of the breast [10]. Also, low FOXD3 expression predicts poor prognosis in high-grade glioma patients [11]. Further, inhibition of the EGFR-Ras-Raf-MEK-ERK signaling by FOXD3 suppressed colon cancer progression [12]. However, the role of FOXD3 in anaplastic thyroid cancer (ATC) is not clear.

Therefore, we investigated the mechanistic role of FOXD3 in thyroid cancer progression by evaluating ATC cell lines and patient tissue samples. FOXD3 knockdown could lead to abnormal cell proliferation and inhibit cell apoptosis. Subsequently, FOXD3 knockdown could also downregulate the expression of E-cadherin via activation of MAPK/EKR signaling pathway. Clinical studies also showed FOXD3 was high expressed in normal samples campared with tumor samples and its expression was negatively correlated to p-ERK, suggesting that FOXD3 is critical for thyroid tumor genesis and progression.

## RESULTS

### FOXD3 knockdown increased growth and proliferation of ATC cell lines

**Figure 1:**
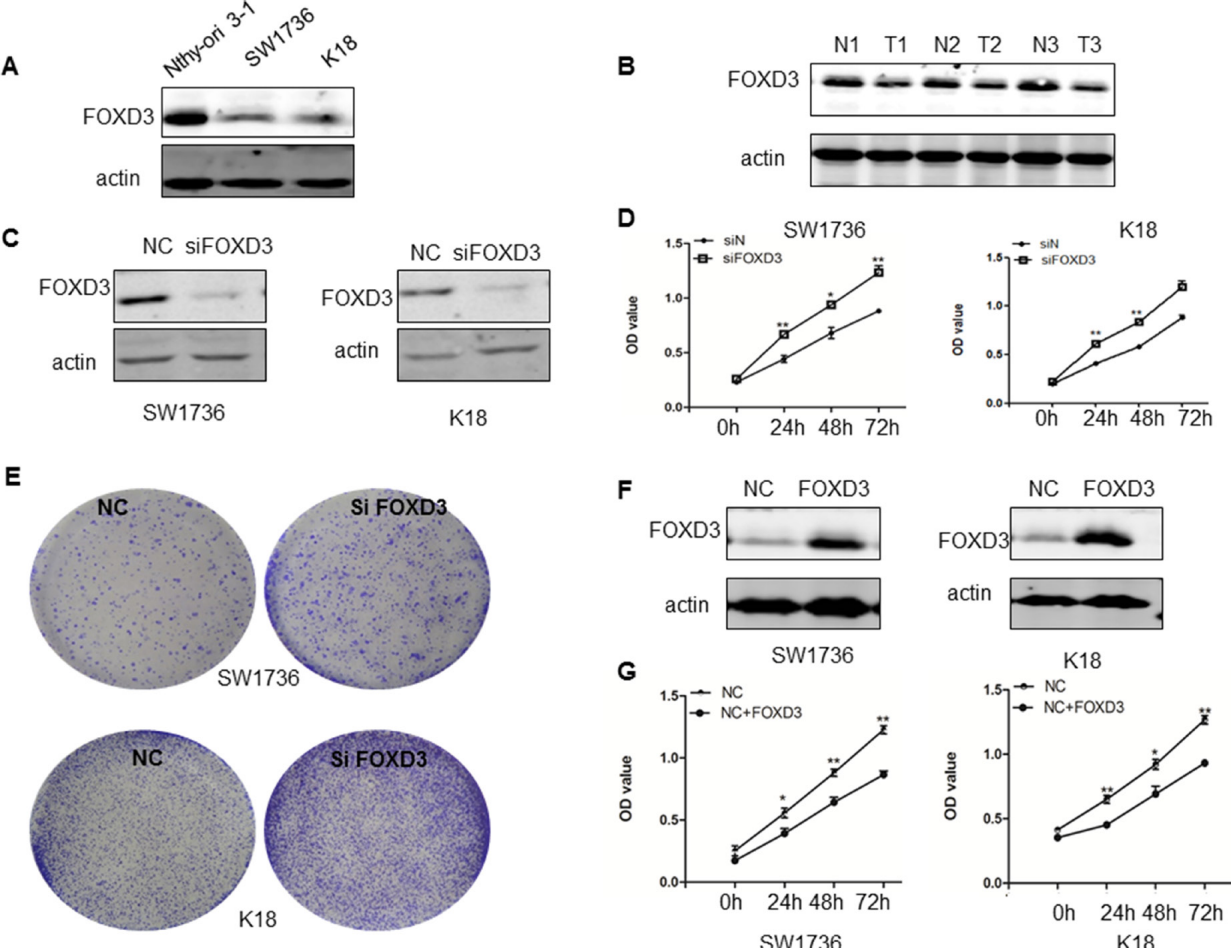
*FOXD3* promotes anaplastic thyroid cancer cell growth and proliferation (**A**) FOXD3 levels in the normal thyroid cell line, Nthy-ori 3-1 and ATC cell lines, SW1736 and K18 were compared by western blot. (**B**) Immunoblot analysis of FOXD3 in normal thyroid and anaplastic thyroid tumor samples (**C**) The control and si*FOXD3* transfected SW1736 and K18 cells were cultured for 72 h and the FOXD3 protein levels were determined by western blot. (**D**) Cell proliferation of control and si*FOXD3* transfected SW1736 and K18 cells was determined at 24, 48 and 72 h by MTT cell viability assay. (**E**) Colony formation ability of control and si*FOXD3* transfected SW1736 and K18 cells was determined by soft-agar colony assay. (**F**) The FOXD3 levels in control and *FOXD3* overexpression vector transfected SW1736 and K18 cells was determined by western blotting. (**G**) Cell proliferation of control and *FOXD3* overexpression vector transfected SW1736 and K18 cells was determined by MTT assays. *p < 0.05, **p < 0.01. Data are represented as mean ± SEM from three independent experiments.

First, we observed low FOXD3 expression in anaplastic thyroid cancer cell lines (SW1736 and K18) compared to the normal thyroid cell line (Nthy-ori 3-1) (Figure 1A). Similarly, we observed decreased FOXD3 expression in thyroid tumor patient tissues compared to normal tissues (Figure 1B).

Next, siRNA knockdown of *FOXD3* in SW1736 and K18 cells enhanced cell growth as determined by MTT (Figure 1D) and crystal violet staining (Figure 1E) assays. Conversely, overexpression of FOXD3 in SW1736 and K18 cell lines decreased cell proliferation as analyzed by MTT assay (Figure 1F–1G). These data together suggested that low FOXD3 expression was associated with thyroid cancer cell growth and proliferation.

### FOXD3 knockdown inhibits apoptosis in ATC cell lines

**Figure 2:**
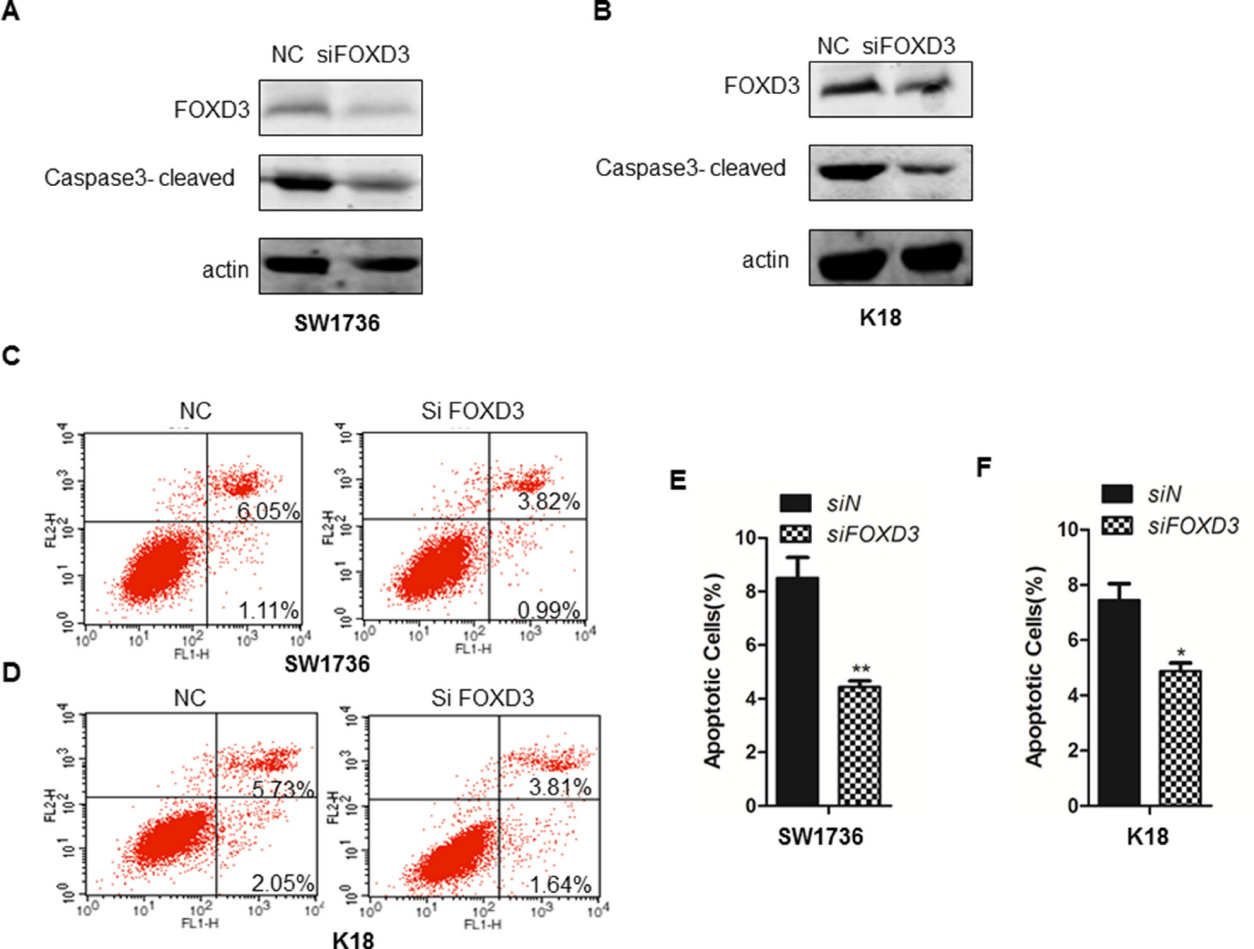
*FOXD3* knockdown inhibits cell apoptosis of ATC cell lines (**A–B**) Western blot analysis of cleaved caspase-3 in control (scrambled siRNA) and *FOXD3* siRNA transfected SW1736 and K18 cells. (**C–D**) Flow cytometry analysis of apoptosis in control (scrambled siRNA) and *FOXD3* siRNA transfected SW1736 and K18 cells, respectively, based on AnnexinV-FITC/Propidium iodide double staining assay. (**E–F**) Quantification of apoptotic cells (AnnexinV^+^ PI^+^) in control (scrambled siRNA) and *FOXD3* siRNA transfected SW1736 and K18 cells.

Next, we analyzed the relationship between FOXD3 expression and thyroid cancer cell apoptosis by analyzing Caspase-3 activity in *FOXD3* silenced SW1736 and K18 cells compared to controls. We observed decreased cleaved caspase 3 in *FOXD3* silenced SW1736 and K18 cells compared to controls (Figure 2A–2B). Further, flow cytometry analysis by AnnexinV/PI double staining revealed that FOXD3 knockdown decreased apoptosis (AnnexinV^+^ PI^+^ cells) in SW1736 and K18 cells compared to controls (Figure 2C–2F). These data demonstrated that low FOXD3 expression decreased apoptosis of anaplastic thyroid cancer cells.

### FOXD3 knockdown promoted invasiveness and EMT of ATC cell lines

**Figure 3:**
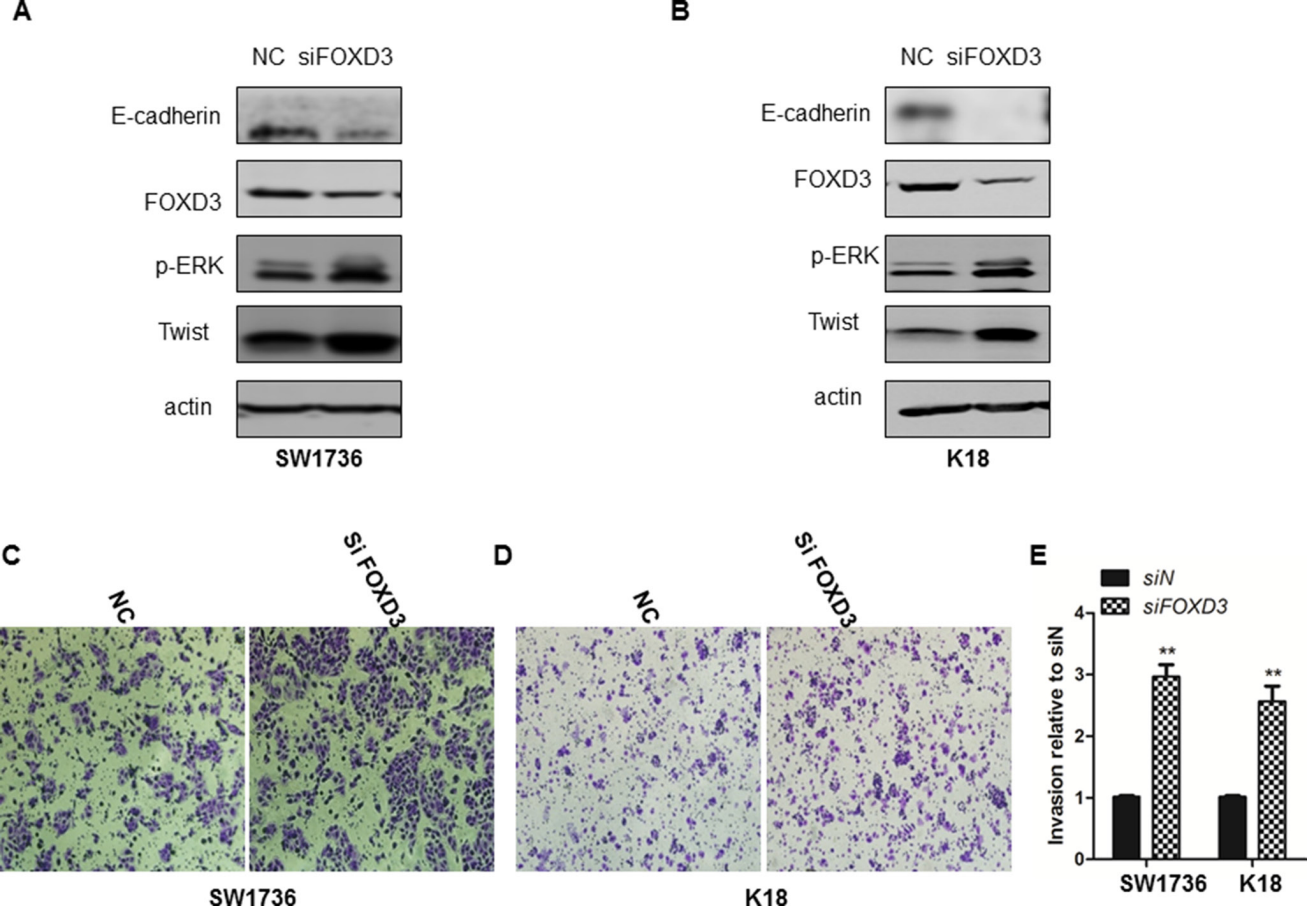
*FOXD3* silencing increased invasiveness and EMT attributes of ATC cell lines (**A–B**) *FOXD3* silencing increased Twist1 and decreased E-cadherin in SW1736 and K18 cells as determined by western blotting. (**C** and **D**) Transwell assay was performed to determine invasiveness of control and si*FOXD3* transfected SW1736 and K18 cells. The control and si*FOXD3* transfected SW1736 and K18 cells were seeded in a Transwell setup and allowed to invade the matrigel for 24 h at 37°C. Magnified images (250X) were captured to show the number of cells that invaded the matrigel. (**E**) Quantification of control and si*FOXD3* transfected SW1736 and K18 cells that invaded the matrigel in the Transwell invasion assay is shown.

We further tested if FOXD3 expression was associated with epithelial-mesenchymal transition (EMT) and metastasis. First, we analyzed expression of TWIST1, E-cadherin and p-ERK that are involved in EMT and metastasis of breast cancer [13]. We observed that *FOXD3* knockdown increased Twist1 and p-ERK levels, but, E-cadherin remained constant and was not downregulated (Figure 3A–3B). In addition, knockdown of *FOXD3* enhanced invasiveness of SW1736 and K18 cells in the Transwell migration assay (Figure 3C–3E). This suggested that low FOXD3 enhanced EMT and metastasis of anaplastic thyroid cancer cells.

### FOXD3 downregulation enhances MAPK/ERK signaling

**Figure 4:**
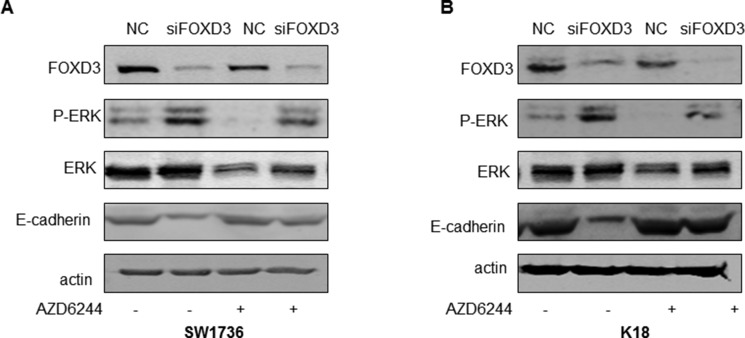
*FOXD3* regulates MAPK/ERK signaling and EMT in ATC cell lines (**A–B**) Western blot analysis of p-ERK and FOXD3 in si*FOXD3* transfected SW1736 and K18 cells treated with or without MEK inhibitor (AZD6244) is shown.

Since the MAPK pathway is upregulated in thyroid cancer because of activating *BRAF* mutations and others, we evaluated the effects of *FOXD3* knockdown on ERK1/2. Western blot analyses showed that transient *FOXD3* knockdown markedly increased the p-ERK in SW1736 and K18 cells (Figure 4A–4B). Further, we treated *FOXD3* knockdown SW1736 and K18 with MEK inhibitor AZD6244 and observed that p-ERK levels were normalized (Figure 4A–4B). Together, these results suggested that MAPK/ERK pathway was regulated by FOXD3 in ATC cells.

### FOXD3 knockdown promoted xenograft growth and upregulated ERK1/2

**Figure 5:**
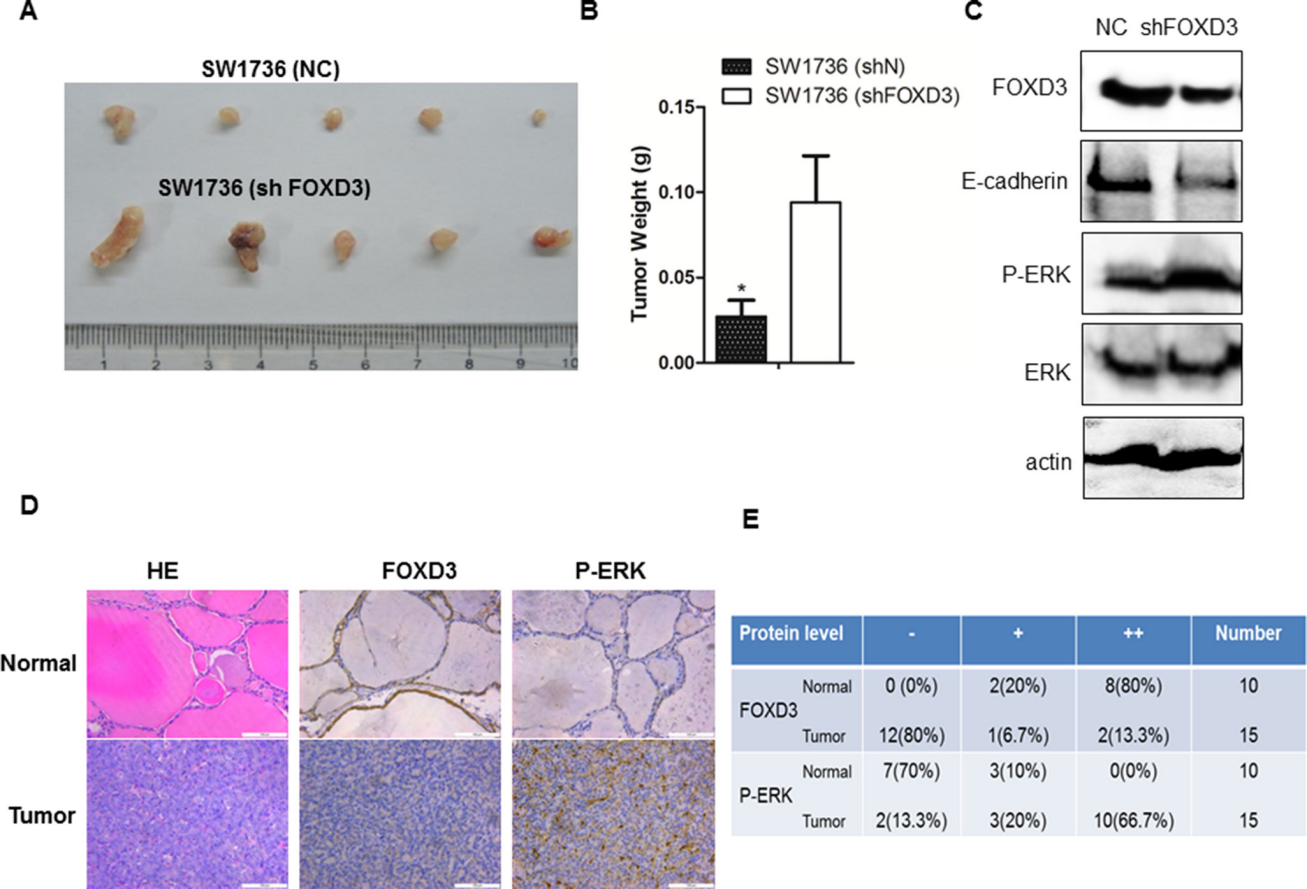
*FOXD3* and MAPK/ERK signaling pathways are inversely correlated in human anaplastic thyroid tumors (**A**) Xenograft tumors were generated by injecting SW1736(ShN) and SW1736 (ShFOXD3) cells into dorsal flanking sites of 5 week old Balb/c nude mice. Tumors were harvested from both groups of mice, four weeks after xenograft. (**B**) The average weight of xenograft tumors from the two groups of mice [SW1736(ShN) and SW1736 (Sh*FOXD3*)] is shown. (**C**) *FOXD3* knockdown increased p-ERK and decreased E-cadherin in mouse xenograft tumors as determined by western blot. (**D**) Immunohistochemical analysis of FOXD3 and p-ERK expression in normal thyroid and anaplastic thyroid cancer patient samples (magnification, 20X). (**E**) Semiquantitative analysis of FOXD3 and p-ERK immunohisochemical staining in normal thyroid and anaplastic thyroid cancer patient samples.

Next, we performed murine xenograft experiments with *FOXD3* knockdown and control SW1736 cells to understand the *in vivo* relevance of FOXD3 in anaplastic thyroid tumor growth. We observed that tumors derived from *FOXD3* knockdown SW1736 cells were larger than those from control SW1736 cells. This was further reflected by concomitant increase in tumor weight from *FOXD3* knockdown SW1736 cells compared to controls (Figure 5A, 5B). Furthermore, reduced FOXD3 protein expression in the knockdown tumor samples was also associated with low E-cadherin and high p-ERK1/2 levels compared with the controls (Figure 5C). Immunohistochemical analysis further revealed low FOXD3 and high p-ERK1/2 expression in human anaplastic thyroid tumors compared to normal tissue samples (Figure 5D–5E), thereby confirming the association between FOXD3 and MAPK/ERK signaling.

## DISCUSSION

Although significant progress has been made in the treatment options available for advanced thyroid cancers, the survival rates remain extremely poor. Hence, identification of new therapeutic targets is of paramount importance. Thyroid cancer is also highly heterogeneous. Anaplastic thyroid cancer is rare undifferentiated form of thyroid cancer that accounts for 1 to 2% of all thyroid cancer cases. In this study, we postulated that the FOXD3 transcription factor may play a significant role in analplastic thyroid cancer growth based on studies in other cancers. Our study confirmed that low FOXD3 levels were associated with ATC growth and metastasis. Previously, low FOXD3 levels were observed in human neuroblastoma tissues and cell lines suggesting that FOXD3 played a tumor suppressor role [14]. Further, activation of miR137 transcription by FOXD3 inhibited progression of human hepatocellular carcinoma [15]. It also suppressed growth and progression of neuroblastoma cells by directly regulating the transcription of NDRG1 [14]. Elevated FOXD3 significantly inhibited gastric cancer progression [16–18]. In melanoma cells, ectopic induction of FOXD3 resulted in G1/S phase arrest and suppressed tumor migration and invasion [19]. Also, FOXD3 deficiency promoted breast cancer EMT and lymph node metastases [20]. Our study demonstrated that FOXD3 knockdown increased human anaplastic thyroid cancer cell proliferation and enhanced invasivess and EMT attributes and reduced cancer cell apoptosis. Moreover, silencing *FOXD3* in human ATC cells enhanced MAPK/ERK signaling. Thus, we concluded that FOXD3 suppressed human thyroid tumorigenesis by regulating MAPK/ERK signaling pathway.

Anaplastic thyroid cancer is an aggressive, rapidly metastasizing tumor with poor prognosis. We observed that FOXD3 deletion increased invasiveness of SW1736 and K18 cell lines and decreased E-cadherin expression. These results indicated that FOXD3 regulated human ATC metastasis, which is the main cause of patient death. A previous study showed that FOXD3 expression decreased cancer cell migration that was efficiently reversed by TWIST1 overexpression [8]. Also, FOXD3 expression in melanoma cells downregulated migration and invasion by inhibiting Rnd3 expression [8, 21], consistent with our results. Thus, we postulate that FOXD3 is a repressor of cancer metastasis. Further studies are required to determine if overexpression of FOXD3 in human ATC cells could inhibit their aggressive character and reverse the expression of thyroid specific genes. Moreover, the detailed mechanistic role of FOXD3 as a tumor suppressor in ATC needs to be ascertained.

The RAS family members regulate RAF–MEK–ERK signaling pathway, which has been implicated in many tumors including ATC [22]. Also, RAS mutations have been reported in benign and malignant thyroid tumors [23]. BRAF is a serine/threonine kinase that regulates cell proliferation through MEK/ERK signaling pathway and is the most common mutation in papillary thyroid cancer. In ATC, BRAF mutations vary from 0 to 50% [24]. Also, Abel *et al*. demonstrated that mutant BRAF suppressed FOXD3 expression [19]. Therefore, ERK1/2 activity is critical in human thyroid carcinogenesis. In this study, we found that FOXD3 deficiency was associated with enhanced ATC cell proliferation, invasion and migration coupled to activation of ERK1/2, which could be reversed by ERK1/2 inhibitor. Hence, we conclude that FOXD3 regulates ATC partly through its modulation of MAPK/ERK signaling pathway. Further investigations are necessary to reveal interactions between FOXD3 and ERK in human ATC.

In conclusion, we demonstrated that *FOXD3* knockdown resulted in enhanced ATC proliferation, invasion and migration and diminished cellular apoptosis. Further, we showed that FOXD3 regulated expression of E-cadherin by modulating MAPK/EKR signaling pathway that promotes EMT and metastasis during thyroid carcinogenesis.

## MATERIALS AND METHODS

### Cell culture of ATC cell lines

Nthy-ori-3-1, SW1736 and K18 were purchased from ATCC. The stable FOXD3 knockdown cell lines were generated by transfection of retroviral shRNA vectors with FOXD3 or control shRNA obtained from OriGene (Rockville, MD).

### RNA interference and RT-PCR analyses

For transient transfections, the following siRNA against FOXD3 was used: 5-GCAAUAGGGACGCGCCAAU-3 [21]. To analyze by RT-PCR, total RNA was extracted from SW1736 and K18 cells and 2 μg RNA from the samples was reverse transcribed into cDNA with M-MLV reverse transcriptase (Invitrogen) following the manufacturer's instructions. The primers for FOXD3 were as follows: forward, 5-AGCAAGCCCAAGAATAGC-3; reverse, 5-TC CAGGGTCCAGTAGTTG-3.

### MTT cell viability and soft agar colony assays

For cell viability assay, cells were cultured in 24-well plates at 37°C in a CO2 incubator after transient transfection with FOXD3 or control siRNA for 24, 48 and 72 h, respectively. This was followed by incubation with 50 μl/well of 10 mg/ml 3-(4,5-dimethyl-thiazol-2-yl)-2,5-diphenyltetrazolium bromide (MTT) at 37°C for 2 h. Then, the supernatant was aspirated, and the MTT-formazan crystals were dissolved in 500 μl of DMSO. The absorbance was measured at 570 nm by a microplate reader.

For soft agar assay, 5 × 10^4^ cells per well were seeded in triplicates into 96 well dishes and cultured at 37°C for 7 days. Individual colonies were fixed and stained with 0.2% crystal violet in 10% ethanol for 30 minutes. The amount of dye taken up by the monolayer was quantified in a spectrophotometer or plate reader at 570 nm [12].

### Apoptosis assays

The control and FOXD3 knockdown cells were harvested, washed twice with pre-chilled PBS and resuspended in 100 μl 1X binding buffer by Annexin V-FITC/PI Apoptosis Detection Kit (BD, 556547). Then, 5 μl annexin V-FITC and 5 μl PI were added to the cell suspension and incubated for 15 min followed by addition of 400 μl 1× binding buffer. The cells were analyzed in a Beckman Coulter FC500 flow cytometer [12] and the AnnexinV^+^ PI^+^ double positive cell numbers were determined by analyzing the FACS data.

### Western blot

Western blot analysis was performed to determine the effects of FOXD3 silencing on MAPK signaling pathway and EMT transition in thyroid cancer cells and tumor tissues [12]. ATC cells were washed with cold PBS and lysed with 1%NP40 and the supernatant was used as total protein lysate. On the other hand, human thyroid tumor samples were homogenized in liquid nitrogen and lysed for 30 min in ice-cold protein extraction buffer. Equal amounts of total protein (50 μg) were separated on a 10% SDS-PAGE and transferred onto nitrocellulose membrane. Then, after blocking, the membranes were incubated with primary monoclonal antibodies overnight at 4°C. The following primary antibodies were used: FOXD3 (Millipore), β-actin (Sigma), TWIST1 (Proteintech) and p-ERK, ERK, E-cadherin and cleaved caspase-3 (Cell signaling). Next, the membrane was incubated with fluorescent labeled secondary antibody for 1 h. Finally, the protein bands were visualized and quantified using the Odyssey Infrared Imaging System (LI-COR, USA).

### Immunohistochemistry (IHC)

For immunohistochemical (IHC) analysis, ATC tumor tissues were fixed with 4% paraformaldehyde for 3 days and embedded in paraffin and 4 μm thick sections were cut. Then, the paraffin sections were dewaxed in xylene and rehydrated with graded concentrations of ethanol and stained with hematoxylin and eosin. Then, the sections were incubated with primary antibody overnight at 4°C and further developed with Histostain-Plus (DAB) IHC kit (Mingrui Biotech, Shanghai) [12].

### Mouse xenograft experiments

Five week old female BALB/c nude mice were bought from Shanghai Silaike Experimental Animal Co.,Ltd. Then, 2 × 10^6^ SW1736 cells in 100 μl PBS were implanted into the dorsal flanking sites of nude mice. Four weeks after injection, the mice were sacrificed and the tumors were harvested and their size and weight assessed. Samples from the tumors were also subjected for western blotting assay to estimate FOXD3, E-cadherin and p-ERK.
